# Integrating the Interleukins in the Biomarker Panel for the Diagnosis and Prognosis of Patients with Acute Coronary Syndromes: Unraveling a Multifaceted Conundrum

**DOI:** 10.3390/diagnostics15101211

**Published:** 2025-05-11

**Authors:** Amalia-Stefana Timpau, Egidia-Gabriela Miftode, Irina-Iuliana Costache-Enache, Antoniu-Octavian Petris, Ionela-Larisa Miftode, Ivona Mitu, Radu-Stefan Miftode

**Affiliations:** 1 Department of Internal Medicine I (Cardiology), Faculty of Medicine, University of Medicine and Pharmacy “Grigore T. Popa”, 700115 Iasi, Romania; darieamalia@gmail.com (A.-S.T.); irina.costache@umfiasi.ro (I.-I.C.-E.); antoniu.petris@umfiasi.ro (A.-O.P.); radu-stefan.miftode@umfiasi.ro (R.-S.M.); 2 Department of Infectious Diseases, Faculty of Medicine, University of Medicine and Pharmacy “Grigore T. Popa”, 700115 Iasi, Romania; egidia.miftode@umfiasi.ro; 3 Department of Morpho-Functional Sciences II, University of Medicine and Pharmacy “Grigore T. Popa”, 700115 Iasi, Romania; ivona.mitu@umfiasi.ro

**Keywords:** biomarkers, acute coronary syndromes, IL-10

## Abstract

**Background and Objectives:** Despite the latest advancements in interventional procedures and pharmacological therapy, the incidence of heart failure and death rate following an acute myocardial remain unacceptably high. This study was designed in response to the limited and conflicting literature data regarding the diagnostic and prognostic role of modern inflammatory biomarkers in patients with coronary artery disease. **Materials and Methods:** We conducted a case–control, prospective observational study. A total of 145 patients were analyzed, of whom 105 patients had an acute coronary syndrome diagnosis and represented the study group, while 40 patients with a chronic coronary syndrome diagnosis represented the control group. This study investigates the diagnostic and prognostic role of the interleukin 1β (IL-1β), interleukin 6 (IL-6), interleukin 10 (IL-10), Growth differentiation factor 15 (GDF-15), and classic biomarkers in patients with ischemic coronary heart disease. **Results:** IL-1β exhibited a prognostic role, being significantly correlated with a left ventricular ejection fraction below 30%. GDF-15 plays a dual role, as a cardio-inflammatory biomarker, being significantly correlated with both N-terminal pro-brain natriuretic peptide (NT-proBNP), and IL-1β, IL-6, and CRP. At the same time, GDF-15 represents a surrogate marker for renal dysfunction. According to the ROC analysis, patients at high mortality risk can be identified with adequate accuracy by cardiac troponin, GDF-15, and IL-10, in addition to NT-proBNP. Logistic regression models confirmed NT-proBNP and IL-10 as mortality predictors. **Conclusions:** IL-1β stands out for its significant prognostic role, while IL-6 did not demonstrate a diagnostic or prognostic role in acute myocardial infarction patients. IL-10 demonstrated superior predictive value in terms of fatal prognosis compared with the other modern biomarkers. GDF-15 is representative of a multivalent biomarker involved in inflammation, heart failure, and renal dysfunction.

## 1. Introduction

Coronary artery disease (CAD) continues to stand out as the leading cause of mortality worldwide, with important regional patterns. While, in developed countries, there is a decreasing trend concerning the incidence and mortality associated with CAD, in low- or middle-income countries, the last few decades have brought a fulminant increase, not only in diagnosed cases, but also in mortality rates and CAD-related disability-adjusted life years (DALYs) [1].

Moreover, although CAD classically affects the elderly population, an epidemiological shift toward younger populations has been recently observed in the context of the global increase in the prevalence of risk factors for atherosclerotic disease, such as diabetes, hypertension, smoking, obesity, and even socioeconomic status [2,3]. These conditions are associated with a hyperinflammatory status and are interrelated with endothelial dysfunction and dyslipidemia, as the central pillars of the pathophysiological triad of atherosclerosis. An activated endothelium enhances the expression of a plethora of adhesion molecules, growth factors, and cytokines that will further lead to local inflammation, fibrosis, intimal hyperplasia, and lipid accumulation, thus creating the framework for the development of atheromatous plaque [4,5].

Another mechanism is related to the substantial platelet activation that is induced by systemic inflammation, via certain interleukins (i.e., IL-1β, IL-6, and IL-8) that bind to the platelets’ surfaces, thus promoting the interaction with an already dysfunctional endothelium. A plethora of prothrombotic molecules, such as interferon-γ (IFN-γ), IL-1β, IL-6, IL-17A, IL-9, chemokine ligand 2, and transforming growth factor-β (TGF-β), doubled by a deficit of nitric oxide (NO), further enhance the process of atherothrombosis, with the subsequent development of myocardial necrosis and acute coronary syndromes (ACSs) [6,7].

## 2. Materials and Methods

Considering the aforementioned molecular aspects, for this study, we conceived a multimarker panel including IL-1β, IL-6, and IL-10, thus aiming to assess the balance between pro- and anti-inflammatory response in patients with acute myocardial infarction (AMI), both with or without ST-segment elevation (STEMI and NSTEMI). Additionally, we included the classic C-reactive protein (CRP), alongside cardiac Troponin I (cTn), N-terminal pro-brain natriuretic peptide (NT-proBNP), and the less-studied Growth/differentiation factor-15 (GDF-15). The rationale behind this approach is that the intensity of the inflammatory response in AMI is directly related to the extension of the infarcted area, thereby being a strong predictor of cardiac remodeling and the subsequent risk of heart failure (HF) [8].

### 2.1. Biomarkers

IL-6 is a ubiquitous, pro-inflammatory cytokine with pleiotropic effects, as it plays a certain role in the development and progression of atheroma plaques, thus potentially destabilizing the atherosclerotic lesions [9,10]. Moreover, IL-6 promotes the initiation and progression of ischemia–reperfusion injury, its low expression correlating with a limitation of infarct size [7]. These molecular findings were clinically validated by multiple studies that confirmed the relationship between elevated IL-6 serum levels and the presence, extension, and severity of CAD [11,12,13]. However, given the rather high inter-individual variability expressed by IL-6, a specific high-risk cut-off is still a matter of debate.

**Figure 1:**
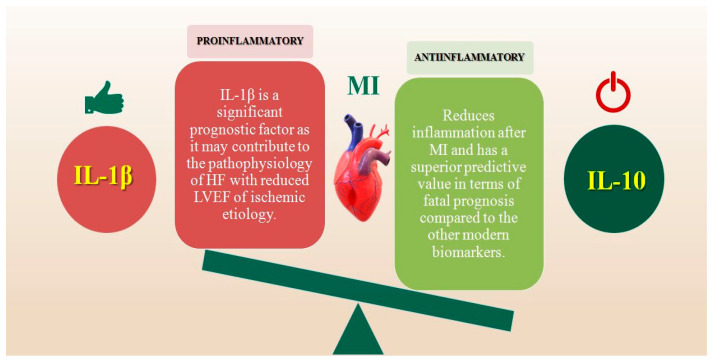
The fragile imbalance between pro- and anti-inflammatory cytokines in patients with acute myocardial infarction. IL-1β—Interleukin 1β, IL-10—Interleukin 10, LVEF—Left-ventricle ejection fraction, HF—Heart failure, MI—Myocardial infarction.

IL-1β is an apical cytokine in the inflammatory cascade that induces IL-6 synthesis. In atherosclerosis, IL-1β promotes the phenotype change of smooth muscle cells, facilitating foam cell formation. During the acute phase of AMI, necrotic cardiomyocytes release IL-1α, while monocytes are a source of IL-1β, as there is a growing body of evidence indicating that increased levels of this cytokine are a predictor for systolic ventricular dysfunction and excessive ventricular remodeling, especially in patients with STEMI (Figure 1) [14,15].

IL-10 is an anti-inflammatory cytokine expressed by several immune cells. The benefits associated with IL-10 have been previously highlighted in experimental studies, with low serum concentrations being correlated with extensive atherosclerotic lesions, doubled by a higher risk of plaque vulnerability and even rupture [7,16]. Despite IL-10 wielding an anti-atherogenic role, even from the initiation of fatty streaks, in human subjects, the IL-10 levels exhibit a highly variable expression pattern, with no specific concentration differences between controls and patients with ACS or stable CAD [7,17,18].

GDF-15 is a member of the TGF-β superfamily, with low concentrations being physiologically synthesized in most tissues. However, increased levels of GDF-15 have been constantly detected in patients with cardiovascular diseases, with this molecule emerging as a biomarker associated with myocardial inflammation and fibrosis. Although it plays a cardioprotective role, having antioxidant, anti-inflammatory, and antiapoptotic effects, recent data suggest that steadily increased GDF-15 could be used as a predictor of poor outcomes in patients with AMI or heart failure [19,20].

We hypothesized that increased levels of IL-6, IL-1β, IL-10, and GDF-15 in patients with AMI correlate with left-ventricular ejection fraction, coronary lesion extension, and short-term prognosis. The main aim of this study is to characterize the inflammatory reaction associated with AMI and to evaluate the diagnostic and prognostic role of the modern inflammatory and cardiac biomarkers IL-6, IL-10, IL-1β, and GDF-15 as compared with the classical inflammatory and cardiac biomarkers CRP, NT-proBNP, and cTn. We assessed the predictive role of biomarkers for both the risk of death and the short-term incidence of HF. Conflicting results in the literature regarding the levels of these biomarkers among patients with AMI or CCS and their role prompted the design of this study protocol.

### 2.2. Study Design

We conducted an observational case–control study, which included consecutively enrolled patients hospitalized between March and December 2022 in the Cardiology Department of the largest regional Clinical Emergency Hospital in North-East Romania.

### 2.3. Study Population

**Figure 2:**
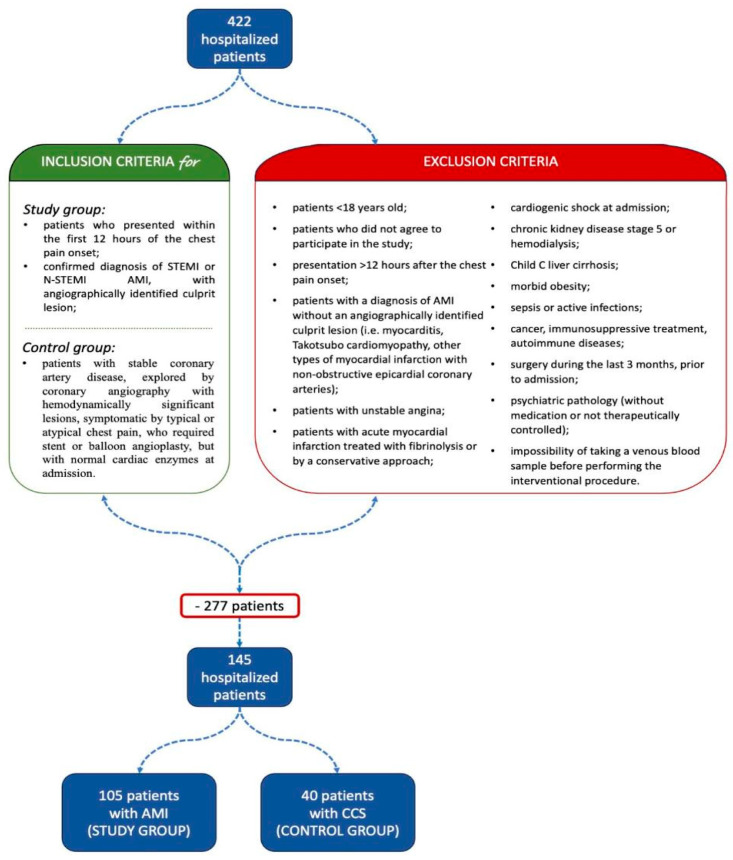
Study design (with inclusion and exclusion criteria).

Preliminary eligibility was evaluated for 422 patients hospitalized during the mentioned period. Using strictly defined inclusion and exclusion criteria (Figure 2), we selected 145 patients who underwent the study protocol. Subsequently, we consecutively enrolled:

- One-hundred and five patients diagnosed with type 1 AMI, representing the target group;
- Forty patients diagnosed with chronic coronary syndromes (CCSs), representing the control group.

AMI was defined according to the Fourth Universal Definition of Myocardial Infarction [21]. Chronic coronary syndromes comprise the clinical phenotypes of a symptomatic patient with reproducible stress-induced angina or ischemia with epicardial obstructive CAD, and the non-acute patients post-ACS or after a revascularization, as stated in the 2024 ESC Guidelines for the management of chronic coronary syndromes [22].

### 2.4. Diagnostic Tests

At admission, all patients underwent a standard clinical examination, doubled by a 12-lead ECG and a complete laboratory test panel (including hemogram, liver and kidney function, and cardiac biomarkers, such as NT-proBNP and troponin). In order to avoid certain bias, we included, in the statistical analysis, the clinical variables assessed strictly at admission, such as blood pressure, heart rate, or body mass index. Echocardiography was performed with a GE Vivid V7 ultrasound system (General Electric, Boston, MA, USA).

Coronary angiography was performed within the first 2 h of first medical contact for patients with STEMI and N-STEMI with severity criteria, respectively, and within the first 24 h in patients with N-STEMI without severity criteria. Coronary angiography was performed using a Philips Azurion 7^®^ angiograph (Philips Medical Systems Nederland, Best, The Netherlands) by physicians trained in interventional cardiology using Judkins diagnostic catheters via radial access as the first choice. Percutaneous transluminal angioplasty (PTCA) was performed with pharmacologically active stents; all patients received guideline-directed medical therapy consisting of pre-procedural dual antiplatelet therapy (DAPT) in loading doses (Aspirine 300 mg plus either Ticagrelor 180 mg or Clopidogrel 600 mg), plus maximal potent statin dose (Atorvastatin 80 mg or Rosuvastatin 40 mg). Angiographic parameters, such as the location of the culprit injury, extension of coronary lesions, number of implanted stents, post-angioplasty TIMI flow, and the need for an aorto-coronary bypass intervention, were included in the database.

A 5 mL venous blood sample for biomarker (IL-1β, IL-6, IL-10, and GDF-15) dosage was collected before angioplasty, within the first 2 h of admission, and then centrifuged for 20 min at 2000 rpm to separate the serum. Subsequently, the serum was divided into 2 Eppendorf tubes that were frozen at −80 degrees until the day of determination in Thermo Scientific Revco Ultima Plus (Thermo Fischer Scientific, Waltham, MA, USA)-model low-temperature freezers. Quantification of IL-6 was conducted using an ELISA kit ab178013 (Abcam, Cambridge, UK) by the ELISA sandwich method. Enzyme immunoassay steps were performed according to the instructions provided in the kit. Quantification of IL-1β and IL-10 was performed using ELISA kits ab214025 and ab46034 (Abcam, Cambridge, UK) using the ELISA Sandwich method. Determination of GDF-15 was performed using EIAab ELISA kits (EIAAB Science Inc., Wuhan, China).

### 2.5. Statistical Analysis

The data were statistically processed using the SPSS Statistics v.23 program (Statistical Package for the Social Sciences, IBM, Armonk, NY, USA). In descriptive analysis, continuous variables were expressed as means (with standard deviations [SD]). Categorical variables were presented as numbers and percentages. For the comparative analysis of the two groups, parametric (*t*-test) or non-parametric (Mann–Whitney U) tests were used. The normal distribution of the variables was assessed using the Kolmogorov–Smirnov test. Pearson and Spearman correlation coefficients (r) were used to assess the correlation between the two variables. To identify the predictors of a phenomenon, we used multivariate logistic regression. The diagnostic performance of the studied biomarkers was assessed by receiver operating characteristic (ROC) analysis and subsequent comparison of areas under the curve (AUCs). Cut-off values were extracted from the ROC curves using statistically validated criteria, such as the Youden Index. The value of 0.05 was used as the threshold of statistical significance.

## 3. Results

The gender analysis revealed a higher prevalence of male patients, with only one-third of the participants being women (Table 1). To avoid biases in the analysis, the age differences between the two patient groups were negligible. The average length of stay was significantly longer for patients with AMI compared with the control group.

Concerning the clinical parameters upon admission, the AMI group exhibited significantly lower systolic and diastolic blood pressures, yet a notably higher heart rate. Chest pain was the most common symptom in both patient groups at admission.

A comparative analysis of inflammatory biomarkers indicated statistically significant inter-group differences concerning IL-1β and IL-6 (Table 2). Regarding cardiac biomarkers, the study group presented elevated mean levels of troponin, CK, CK-MB, and NT-proBNP.

**Figure 3:**
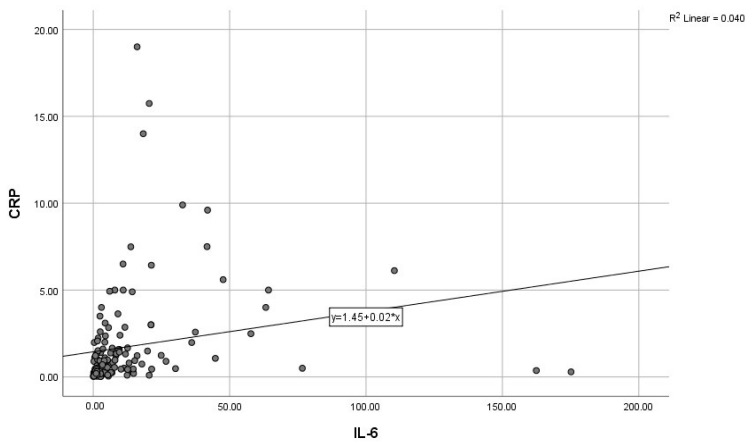
Positive correlation between CRP and IL-6 (CRP—C-reactive protein, IL-6—Interleukin 6).

**Figure 4:**
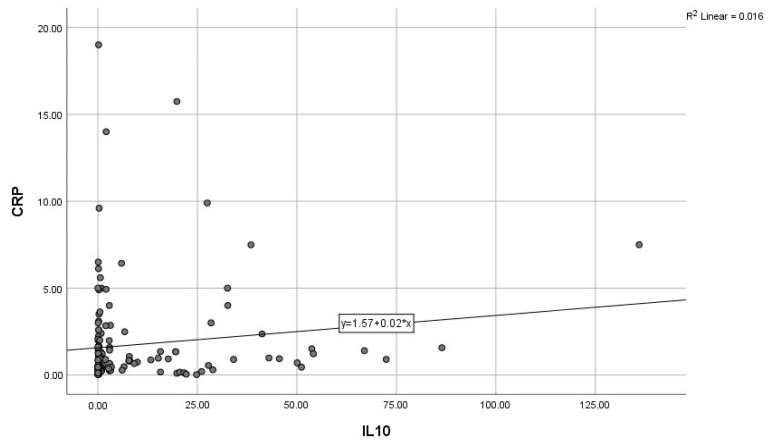
Positive correlation between CRP and IL-10 (CRP—C-reactive protein, IL-10—Interleukin-10).

**Figure 5:**
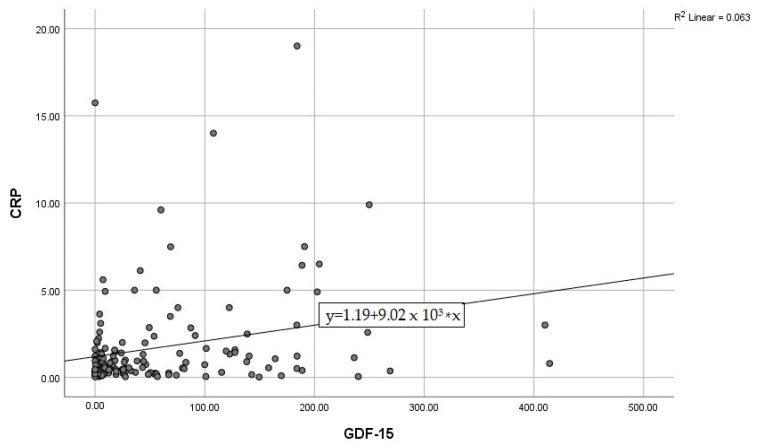
Positive correlation between CRP and GDF-15 (CRP—C-reactive protein, GDF-15—Growth differentiation factor 15).

**Figure 6:**
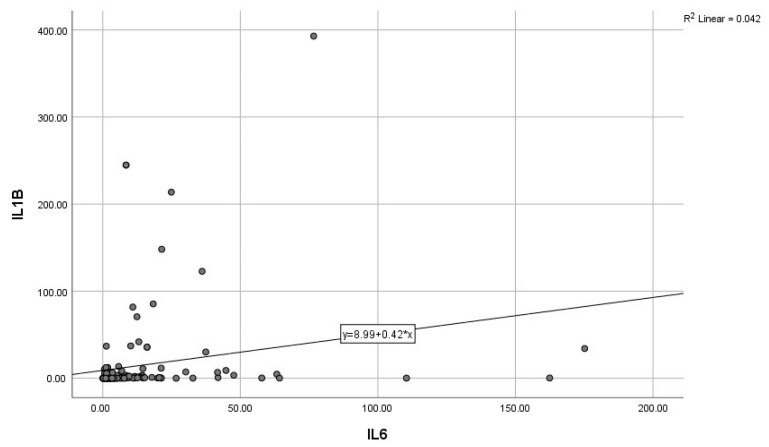
Positive correlation between IL-6 and IL-1B (IL-1β—Interleukin 1β, IL-6—Interleukin 6).

**Figure 7:**
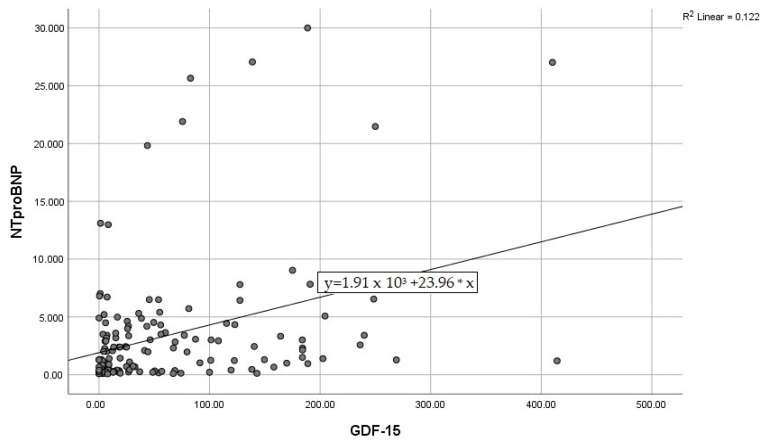
Positive correlation between NT-proBNP and GDF-15 (NT-proBNP—N-terminal pro-brain natriuretic peptide, GDF-15—Growth differentiation factor 15).

**Figure 8:**
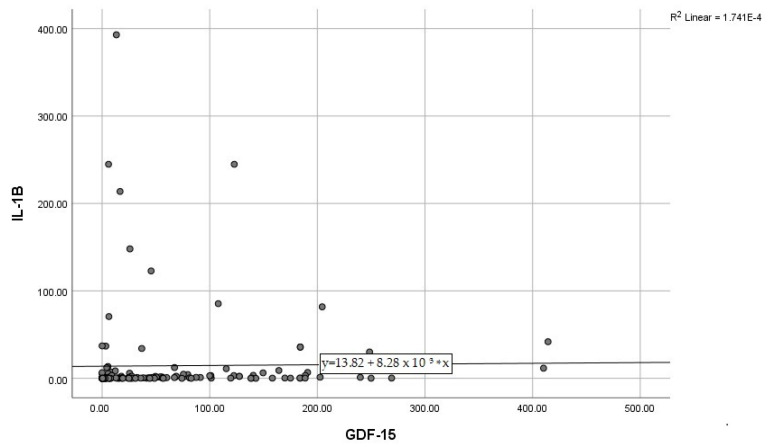
Positive correlation between IL-1B and GDF-15 (IL-1β—Interleukin 1β, GDF-15—Growth differentiation factor 15).

**Figure 9:**
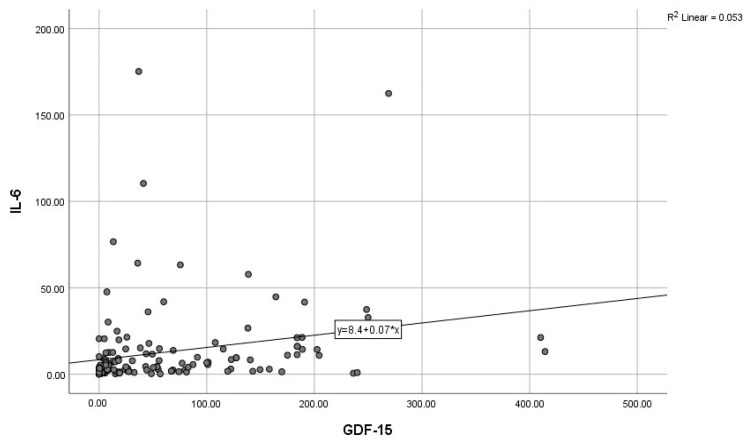
Positive correlation between GDF-15 and IL-6 (GDF-15—Growth differentiation factor 15, IL-6—Interleukin 6).

By calculating correlations between the “classic” CRP, and the modern biomarkers, we observed significant associations of CRP with IL-6, IL-10, and GDF-15 (Figure 3, Figure 4 and Figure 5). Subsequently, we assessed the relationships between modern inflammatory biomarkers, observing that proinflammatory cytokines with interdependent production (IL-1β and IL-6) exhibited a noteworthy positive correlation (Figure 6). GDF-15 plays a dual role, as a cardiac biomarker, significantly correlated with NT-proBNP (Figure 7), as well as an inflammatory biomarker, correlated with IL-1β, IL-6 (Figure 8 and Figure 9), as well as with the previously mentioned CRP.

The most common site of coronary artery occlusion (culprit lesion) was the proximal level of the anterior descending artery (ADA). Comparative analysis of coronary lesions extension showed an increased prevalence of multivascular lesions in patients presenting with AMI. As a result, 69.5% of them had coronary lesions that were subsequently addressed to PTCA after the acute phase of the disease; 96.2% of patients underwent successful revascularization, resulting in a TIMI 3 flow following the deployment of pharmacologically active stents. However, in the other cases, the interventional approach failed due to hard atheroma plaques or distal embolization, resulting in the “no-reflow” phenomenon.

No significant correlation was observed between the site of the culprit lesions in patients with AMI (or the stenosis that was electively stented in patients with CCS) and IL-1β, IL-6, IL-10, GDF-15, or CRP levels (Supplementary Material—Table S1). As a result, we can state that the site of the infarcted area had little to no influence on the serum concentration of inflammatory biomarkers.

Further, we aimed to identify predictors of HF with severely reduced left-ventricle ejection fraction (LVEF). Thus, from a biological perspective, NT-proBNP and uric acid were significantly associated with a severely reduced LVEF (Table 3). Furthermore, a strong inverse significant correlation between LVEF and IL-1β levels was noted.

The Pearson correlation further validated the relationship between NT-proBNP, uric acid, and a severely reduced LVEF (<30%). A high urea serum concentration was the sole renal function parameter that demonstrated a strong association with a significantly impaired LVEF (Table 4). This correlation analysis was performed in order to better assess which biochemical marker to further include in a logistic regression model.

To evaluate the capacity of the cardiac and inflammatory biomarkers in predicting HF with LVEF < 30%, we created a logistic regression model. It showed that increased levels of CRP, uric acid, and NT-proBNP were predictors of a severely reduced LVEF (Table 5).

The regression model was validated using the Hosmer–Lemeshow test, with *p* > 0.05, which indicates that the estimated model is suitable for the analyzed data (Table 6).

The predictive role of biomarkers concerning fatal prognosis was assessed, with IL-10 being directly and significantly related to in-hospital death (Table 7).

The *t*-test, showing a positive and significant correlation between IL-10 and in-hospital mortality, further supported this relationship (Table 8). Additionally, IL-1β exhibited a strong negative correlation with mortality.

**Figure 10:**
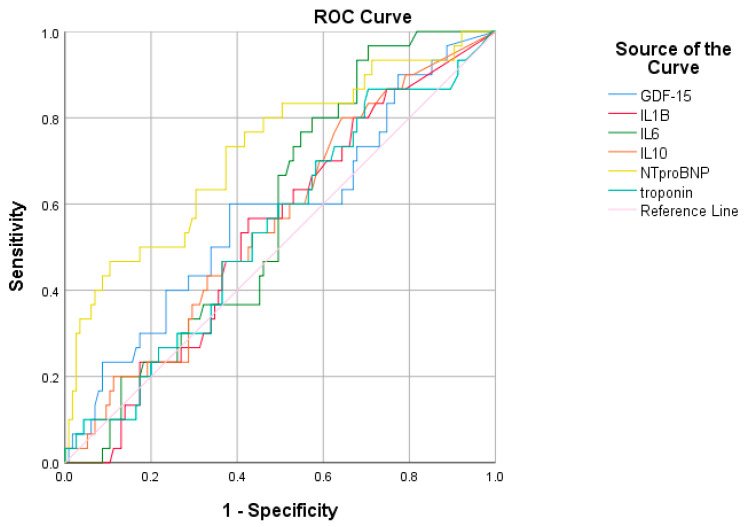
ROC curve for the relationship between biomarkers and a severely reduced ejection fraction (<30%). (IL-1β—Interleukin 1β, IL-6—Interleukin 6, IL-10—Interleukin 10, GDF-15—Growth differentiation factor 15, CRP—C-reactive protein, NT-proBNP—N-terminal pro-brain natriuretic peptide).

We subsequently aimed to assess if other biomarkers were significantly correlated with in-hospital mortality. Thus, IL-10, GDF-15, and NT-proBNP were identified as significant mortality predictors (Table 9). Concerning the prediction value for a reduced ejection fraction (LVEF < 30%), NT-proBNP is still the gold-standard, with a significant AUC of 0.724, followed by GDF-15 (AUC = 0.578), which is superior even to troponin (AUC = 0.543) (Figure 10). On the other hand, the other inflammatory biomarkers, albeit superior to troponin, exhibited a rather limited predictive value for a decreased ejection fraction when compared with NT-proBNP and cardiac troponin.

The biomarkers that were significantly correlated with in-hospital mortality (IL-10, GDF-15, and NT-proBNP) were included in a multivariate logistic regression model. Although correlated per se with fatalities, GDF-15 was no longer a predictor of mortality in the multimarker model (Table 10).

However, a dual NT-proBNP&GDF-15 assessment is a superior (albeit not statistically significant) predictor model compared with each biomarker individually (Table 11).

Somewhat predictable correlations were also found between mortality rate and a LVEF < 30%, or NYHA functional class (Table 12). Parameters such as the need for inotropic support and orotracheal intubation, previously correlated with LVEF < 30%, were also significantly correlated with in-hospital death, serving as additional poor prognostic factors.

We also developed a non-biomarker prediction model, based on NYHA functional class, oxygen therapy, inotropic support requirement, and LVEF < 30% (Table 13). In the multimarker model, only orotracheal intubation and LVEF < 30% remained relevant, the two parameters being substantially correlated with mortality. According to this model, a mortality of over 70% can be predicted in patients with LVEF < 30% and orotracheal intubation.

By excluding variables that no longer contributed to the predictive score, we obtained a simpler model where only orotracheal intubation and NT-proBNP remained significant mortality predictors in the clinical–biological score (Table 14).

**Figure 11:**
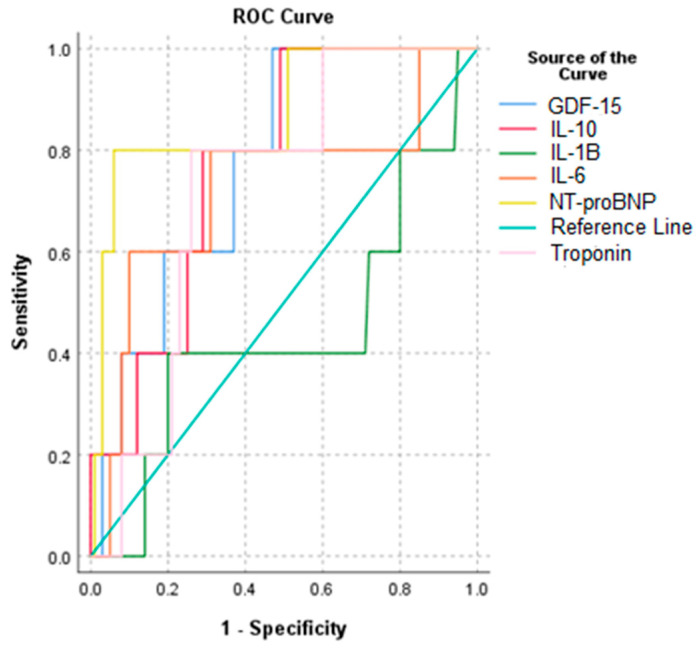
ROC curve for the relationship between biomarkers and mortality.

We conducted a ROC analysis to assess biomarkers’ performance in predicting a fatal prognosis (Table 15). It revealed that IL-10, GDF-15, and cTn can accurately identify patients at high risk of death, while NT-proBNP performs the same role with even greater accuracy (Figure 11).

IL-1β—Interleukin 1β, IL-6—Interleukin 6, IL-10—Interleukin 10, GDF-15—Growth differentiation factor 15, NT-proBNP—N-terminal pro-brain natriuretic peptide.

## 4. Discussion

From a demographic perspective, there were no statistically significant variations regarding age between the two groups. Although chronic systemic inflammation is commonly associated with aging, in our study, the absolute age did not influence the results [23]. Concerning the gender distribution, we noted a higher proportion of male patients in both groups. Studies across different continents consistently report that men are more prevalent among patients with AMI [24,25]. However, regardless of clinical characteristics, women are found to have a higher risk of AMI recurrence, but not of developing complications, such as HF or even death [24].

Classic inflammatory biomarkers (leukocytes, CRP, and ferritin) and modern ones (IL-1β, IL-6, and IL-10) were analyzed in the present study to provide a comprehensive overview of the inflammatory response associated with AMI. Compared with the control group, we noted considerably increased levels of all modern biomarkers in AMI patients. AMI is linked to a complex inflammatory response that might have a bidirectional relationship with inflammation. An inflammatory response results from the occlusion of a coronary artery, but a ubiquitous inflammatory status can also lead to plaque instability, rupture, and subsequent thrombosis [26,27].

One major objective of this study was to assess the prognostic role of biomarkers in predicting short-term HF following AMI. We approached the “cytokine theory” (important not only in infectious diseases like COVID-19 [28], but also in cardiovascular conditions) in HF following AMI, focusing on studying the relationship between inflammatory molecules, cardiac biomarkers, and systolic performance (expressed as LVEF). In this regard, GDF-15 was correlated with CRP, IL-1β, and IL-6, as well as with NT-proBNP, the primary biomarker in HF diagnosis. In contrast, we found no correlation between inflammatory biomarkers and NT-proBNP levels. As a result of its correlation with inflammatory and cardiac biomarkers, GDF-15 confirmed its potential dual role as an early, subclinical predictor of myocardial injury or inadequate immune response. Previous studies have described GDF-15 not solely as a proinflammatory molecule [19,29,30].

During the acute phase of AMI, the myocardium is initially infiltrated by neutrophils [31]. We observed that total leukocyte and neutrophil counts were significantly higher in patients with AMI compared with those with CCS. The resolution of the local inflammatory process is mediated by the action of anti-inflammatory cytokines, with interleukin-10 (IL-10) playing a pivotal role. Through interactions with immunoregulators like interleukin-2, IL-10 modulates the interplay between T cells and macrophages, fostering myocardial healing [32]. In the included patients, the levels of IL-10 exhibited a direct and significant correlation with CRP values, indicating the immune system’s endeavor to rebalance its pro- and anti-inflammatory components. Research by Singh et al. illustrated that CRP inhibits IL-10 synthesis in vitro, and the administration of CRP to mice with AMI led to a notable reduction in IL-10 levels. By diminishing the IL-10 levels, CRP disrupts the equilibrium between anti-inflammatory and pro-inflammatory cytokines, triggers an inflammatory state, and plays a central role in atherothrombosis and the extension of the infarcted area [33,34]. An animal model study demonstrating that the absence of endogenous IL-10 accelerates the expansion of the infarcted area highlighted the crucial role of endogenous IL-10. Additionally, Krishnamurthy et al. demonstrated that, by diminishing fibrosis and increasing capillary density in the myocardium, IL-10 enhances systolic function and left ventricular remodeling [35]. Despite its reported “cardioprotective” role in AMI patients, elevated baseline IL-10 concentrations emerged as a robust predictor of an unfavorable short-term outcome in the current study. Cavusoglu et al. identified this anti-inflammatory cytokine as a predictor of long-term complications and cardiovascular adverse events in AMI patients [36]. Thus, our findings, supported by previous literature data, contradict the controversial function of elevated IL-10 levels in AMI patients, indicated by Heeschen et al. as a favorable predictor [37,38,39]. Therefore, it can be concluded that IL-10 reflects a pronounced inflammatory state in AMI patients, plays a crucial role in counteracting the inflammatory response, and serves as a reliable biomarker for predicting mortality risk.

The comparative analysis of mean IL-1β values between the two cohorts revealed a significantly higher IL-1β level in the AMI group compared with the control group. However, previous studies presented conflicting results, with some reporting normal or elevated IL-1β levels in AMI patients [40,41]. Recent years have witnessed a growing interest in targeting IL-1β as part of a therapeutic strategy due to its essential role in atherothrombosis. The interruption of myocardial blood supply triggers an intense acute inflammatory response characterized by inflammasome activation and the synthesis of IL-1β and other proinflammatory cytokines in cardiomyocytes and interstitial cells [42,43]. During the subacute phase of AMI, IL-1β is known to exert deleterious effects on left ventricular dilatation and contractility, related to a reduction in β-adrenergic receptor responsiveness [44,45]. Hence, the concept of administering IL-1β inhibitors in patients with AMI during the acute phase to mitigate pathological cardiac remodeling and progression to HF has been proposed. The prognostic significance of IL-1β has also been explored in decompensated HF, which includes ischemic heart disease as an etiology. The results indicated that elevated IL-1β levels have clinical relevance in patients with decompensated HF, with those exhibiting elevated IL-1β levels facing a substantially higher risk of mortality. These data are further supported by the correlation between IL-1β and the NT-proBNP and cTn serum concentrations [46,47]. Similarly, in the current study involving AMI patients, IL-1β exhibited a significant positive correlation with cTn levels and another cardiac dysfunction biomarker, GDF-15. Therefore, IL-1β may emerge as a potential therapeutic target not only in AMI patients, but also in those with decompensated HF.

While an AMI-associated inflammatory reaction is beneficial and essential for myocardial healing and repair to a certain extent, excessive and persistent activation of the inflammatory cascade can lead to maladaptive remodeling of the left ventricle, culminating in HF [48]. Despite the medication that addresses various pathophysiological aspects, including platelet aggregation, vasodilation, neurohormonal mechanisms, and interventional treatment, the in-hospital incidence of HF following AMI ranges from 4 to 28% [49,50,51]. Although HF following an acute coronary syndrome exhibited a decreasing trend, the associated morbidity and economic burden remain significant [52]. These data have prompted a potential shift in the therapeutic paradigm toward modulating inflammation during the acute phase of AMI, aiming to reduce the maladaptive remodeling.

The classic therapeutic paradigm in atherosclerosis is focused on lipid-lowering drugs and antiplatelet agents. However, recent studies turned the spotlight on the inflammation, as shown in the CANTOS trial, in which Canakinumab, a human monoclonal antibody targeting IL-1β, reduced the total CV burden expressed as nonfatal myocardial infarction, stroke, or cardiovascular death [53]. Moreover, CRP and IL-6 were both significantly reduced in the Canakinumab group, and were thus indirectly associated with a better prognosis [2,53]. Other immunomodulators, such as Tocilizumab or Sarilumab, not only decreased the systemic inflammation per se but also exhibited protective myocardial effects, as these molecules mitigated the Il-6-associated deleterious effects by blocking both its soluble and membrane-attached receptor [54]. Additionally, the administration of the monoclonal antibody Anakinra, a recombinant IL-1 receptor antagonist that inhibits both IL-1β and IL-1α, in patients with STEMI reduced mortality and incidence of newly diagnosed HF, compared with a placebo [55].

There is substantial evidence from preclinical and clinical studies indicating that inflammation plays an essential pathophysiological role in the initiation and progression of coronary artery disease. Anti-inflammatory agents lower the residual inflammatory risk that persists in the late stages of AMI, as the CANTOS trial showed [21,56]. In the VCU-ART3 study, Anakinra was administered to patients with ST-elevation AMI (STEMI) within the first 12 h after the onset of the acute event, then every 24 h. Reductions in HF-related events and high-sensitivity-CRP levels were observed in the first 14 days after the acute coronary syndrome [54]. Naturally, the following questions arise: which patients are suitable to receive these therapies? What is the best moment to administer the monoclonal antibodies targeting the pro-inflammatory cytokines? And particularly in light of potential negative effects on host immunity, how much do they weigh in the risk–benefit balance? These problems are only partially addressed by the available literature, as the topic opens up new research perspectives. Dynamics and the prognostic role of modern biomarkers are of paramount importance in characterizing the AMI-associated inflammatory response and in the therapeutic strategy.

We found an inverse association between LVEF and IL-1β in the included patients. This proinflammatory cytokine may contribute to the pathophysiology of HF with reduced LVEF of ischemic etiology, as evidenced by its involvement in post-infarction ventricular remodeling [14]. Additionally, recent investigations suggest that IL-1β may play a role in the development of diastolic dysfunction and HF with preserved ejection fraction [57,58]. However, another study showed conflicting results and reported non-significantly increased levels of IL-1β in the acute phase of AMI compared with the control group [59]. Such findings may reflect difficulties in detecting plasma IL-1β due to the binding of the cytokine to large proteins, such as α2-macroglobulin, complement, or other soluble receptors [60]; these conflicting data demonstrate the need for further studies investigating IL-1β levels in patients with AMI.

Significantly elevated levels of inflammatory biomarkers, such as IL-1β, IL-6, and CRP, underline the activation of the inflammatory cascade in AMI patients. We discovered significant positive correlations between inflammatory biomarkers, IL-6, IL-1β, and CRP. Moreover, we found a correlation between IL-6 and GDF-15. The latter is a complex cardiac biomarker whose synthesis is triggered by ischemia in cardiomyocytes and whose role is described in the pathophysiology of atherosclerosis [19,61]. IL-6 did not correlate significantly with any of the AMI traditional risk factors. As opposed to findings in the literature, IL-6’s predictive value for mortality or a severely reduced LVEF was not supported by the logistic regression analysis we performed. We observed that, in our patients, IL-6 has predictive value. IL-6 was negatively correlated with LVEF, without reaching statistical significance. In contrast, Groot et al. reported a correlation between a higher IL-6 concentration and a greater infarction area, and correspondingly, a poorer LVEF in patients with STEMI [62]. The findings are further supported by Tiller et al., who observed an independent correlation between increased levels of IL-6 on day 2 following STEMI and decreased myocardial function, increased infarction area extension, and a more severe reperfusion injury [63]. The crucial part that IL-6 plays in the dynamics of post-infarction innate immune activation has been well-described by Huang et al. They highlighted the importance of uncontrolled inflammation on cardiac remodeling, ventricular geometry, and function [64]. Furthermore, Wang et al. showed that, in animal models, suppression of the inflammatory response by lowering proinflammatory cytokine levels in the acute phase of AMI was linked to a reduction in ventricular function [65]. According to these findings, the inflammatory response may prevent cardiomyocyte apoptosis and promote autophagy, which is a physiological mechanism necessary for tissue survival in the early phases of AMI [64,65]. Jing et al., on the other hand, showed that blocking the IL-6-encoding gene improves post-AMI remodeling, most likely through activating macrophages and decreasing collagen synthesis [66]. Additional research using animal models that show the profibrotic function of IL-6 by inducing ventricular hypertrophy and fibrosis by its infusion supports this theory [67]. The inflammatory reaction associated with AMI, which decisively influences post-infarction myocardial remodeling and fibrosis, seems to be a double-edged sword. Despite the controversial results of studies investigating the prognostic role of IL-6 in AMI patients, indicating the existence of still unknown pathways, this proinflammatory cytokine was studied as a therapeutic target [63,66,67,68,69]. A single bolus of Tocilizumab administered during the PTCA procedure has shown promising effects, as demonstrated by the ASSAIL-MI study [69].

This study also aimed to assess if there is a specific location of the coronary occlusion that is associated with higher levels of inflammatory biomarkers. We found no significant correlation in this regard, with biomarker levels being independent of the location of the myocardial necrosis area. Considering the high death rate of HF patients with reduced LVEF one year after diagnosis, we aimed to identify the clinical and paraclinical variables associated with a severely impaired LVEF [70]. We noted a significant correlation between LVEF < 30% and various biological parameters, like NT-proBNP, urea, and uric acid. NT-proBNP is a reliable marker of left ventricular dysfunction, having higher mean values in our study group. The serum uric acid levels in HF patients are associated with increased superoxide dismutase activity, an indicator of oxidative stress. Moreover, uric acid contributes to endothelial dysfunction by lowering nitric oxide production. An additional pathophysiological link between hyperuricemia and HF may be through inflammatory pathways. Data in the literature suggest that even asymptomatic hyperuricemia is associated with a proinflammatory status and increased levels of CRP, IL-6, and neutrophils [71,72]. Furthermore, hyperuricemia correlates with the presence of diastolic dysfunction and a higher incidence of major acute cardiovascular events in patients with AMI, whereas it is not correlated with the degree of coronary stenosis. Correcting hyperuricemia may reduce the incidence of HF and death in AMI patients [72].

Recommendations for long-term management from the 2023 ESC guidelines for the management of acute coronary syndromes indicate that low-dose colchicine, 0.5 mg daily, may be considered if the risk factors are insufficiently controlled or in case of recurrent cardiovascular events under optimal therapy [52]. The literature shows contradicting findings on the effect of colchicine in patients with AMI. On one hand, a recent meta-analysis shows that colchicine usage after MI increases unfavorable gastrointestinal events while decreasing the composite of adverse cardiovascular events and hospitalization urgency. Colchicine has no effect on hs-CRP levels, all-cause mortality, cardiac arrest, stroke, or recurrent MI [73]. In the Colchicine Cardiovascular Outcomes Trial (COLCOT), which included patients with a recent ACS event, low-dose colchicine, demonstrated a significant reduction in cardiovascular death, resuscitated cardiac arrest, MI, stroke, or urgent revascularization compared with a placebo [74].

On the other hand, a recent trial, CLEAR, showed that treatment with colchicine initiated soon after myocardial infarction and continued for a median of three years did not reduce the incidence of the composite primary outcome (death from cardiovascular causes, recurrent myocardial infarction, stroke, or unplanned ischemia-driven coronary revascularization) [75]. A study that hypothesized that colchicine could reduce infarct size and left ventricular remodeling during acute-phase STEMI found that oral administration of high-dose colchicine in the first 5 days after the MI did not reduce infarct size as measured by cardiac magnetic resonance imaging [76]. Nevertheless, additional studies are required to validate these findings.

There were no fatalities recorded among hospitalized patients with CCS, whereas the mortality rate in the study group was 4.7%, a similar result to that reported by McNamara et al. [77]. Our study’s comparatively low mortality rate might be the consequence of excluding patients with severe comorbidities, advanced HF, or a late hospital presentation. Using correlations in statistical analysis, we noted a significant association between the risk of death with GDF-15 and NT-proBNP. However, only NT-proBNP was found to be a mortality predictor when these parameters were added to a multivariate logistic regression model. GDF-15 may be the expression of different pathophysiological pathways involving parietal stress, myocardial injury, or inflammation, exerting a cardioprotective role in the ischemic context, whereas NT-proBNP is synthesized in cardiomyocytes in response to parietal stress, regardless of its etiology [61,78].

We reached our objective of evaluating the predictive performance of modern inflammatory biomarkers in patients admitted to the largest hospital in North-Eastern Romania. Thus, by obtaining an AUC with a high predictive value for NT-proBNP, and an adequate one for IL-10, GDF-15, and cTn, these biomarkers can accurately identify patients at high risk of death. Literature-based data bolster the noteworthy predictive significance of these biomarkers in AMI patients. Higher GDF-15 levels have been linked to a higher risk of cardiac death in the first 24 h after an AMI, according to a Swedish study [79]. Furthermore, in the first month following STEMI, there was a positive correlation observed between higher levels of NT-proBNP and the incidence of major adverse cardiovascular events [78].

The pathophysiology of IL-1β dynamics may account for the absence of significantly elevated cytokine levels in deceased patients. As an “alfa” cytokine that promotes a cascade of inflammatory reactions, its binding to the specific receptor causes a decrease in its plasma concentration [59]. Therefore, it is possible that deceased patients suffered a stronger inflammatory reaction, particularly in the presence of a larger infarction area (as indicated by a higher cTn value) and elevated levels of other inflammatory biomarkers. The increased levels of the anti-inflammatory cytokine IL-10 in non-survivors further support this hypothesis.

We developed non-biomarker prediction models and mixed models incorporating clinical and biological parameters aiming to identify the risk factors contributing to in-hospital mortality. The non-biomarker model highlights the role of LVEF < 30% and orotracheal intubation in predicting death. Since deaths following AMI are not solely related to hemodynamic factors, we additionally developed a clinical–biological regression model that identified NT-proBNP and orotracheal intubation as mortality predictors. Sharma et al. confirmed these findings, while Schellings et al. reported that NT-proBNP has a reliable prognostic role, similar to the GRACE score in 30-day mortality following AMI [80,81]. Among the patients examined, IL-10 is a highly reliable indicator of death. The concomitant elevation of IL-10 levels and their association with elevated CRP levels and mortality may be attributed to the cytokine’s malfunction or incapacity to perform its anti-inflammatory activity in the framework of a hyper-inflammatory state. Using ROC analysis, we observed that NT-proBNP has a strong predictive value for mortality risk in AMI patients, while GDF-15 and cTn have good predictive values. All the biomarkers identified as independent predictors of death might be used for early risk stratification following AMI to improve patient management and prognosis.

The characterization of the inflammatory reaction associated with AMI is the starting point for achieving an important goal of modern cardiology: the development of therapeutic strategies that minimize the area of myocardial necrosis and allow optimal healing of the ischemic myocardium after reperfusion. The existence of the IL-6 receptor blocker, IL-1β antagonist, and their potential to become therapeutic targets to improve the prognosis of patients with AMI increases the importance of characterizing the inflammatory reaction associated with acute myocardial ischemia and coronary reperfusion [82]. Modern rapid reperfusion strategies, antithrombotic therapy, and neurohormonal blockade therapies have substantially reduced the incidence of post-infarction HF in recent decades. However, post-AMI HF remains a public health issue with a direct impact on patients’ quality of life, representing an important economic burden as well [82]. It is possible that the current therapeutic paradigm still overlooks key pathophysiological mechanisms; consequently, depicting the inflammatory reaction in AMI patients could open new research perspectives, potentially turning the studied molecules into therapeutic targets.

Evidence in the literature shows that, whereas the positive impact of IL-10 is widely known, the role of IL-6 is still debatable. IL-6 was not significantly correlated with any of the traditional risk factors for AMI in our study, nor was it linked to either death or a lower LVEF. On the other hand, literature data show that, in patients with STEMI, a higher IL-6 concentration is associated with a larger infarction area and, thus, a lower LVEF [62]. Despite Tocilizumab increasing myocardial salvage in in patients with acute STEMI, as shown by the ASSAIL-MI trial, the final infarct size did not differ significantly between the Tocilizumab and placebo arms [69]. A recent meta-analysis revealed a limited role of IL-6 in cardiac remodeling in animal models with myocardial ischemia, despite the well-established pro-inflammatory role of IL-6 in MI [83]. To ascertain the advantageous effects of IL-6 inhibitors in controlling cardiac remodeling, more fundamental research examining the pharmacological inhibition of IL-6 receptor is needed. Regarding the role of IL-10 in patients with IMA, a large body of research supports its beneficial role. Following MI, IL-10 promotes the improvement of left-ventricle systolic function and the regression of cardiac inflammation. Since it is an anti-inflammatory biomarker, the elevated levels indicate an effort to counteract the hyperinflammatory reaction in the infarcted area. As previously stated, IL-10 may represent a trustworthy biomarker for mortality risk assessment [33,34,35]. The key characteristics concerning the analyzed biomarkers in our study are summarized in Table 16, highlighting their distinct prognostic and diagnostic roles in cardiovascular disease.

Inflammation not only plays a key role in myocardial infarction, but it extends beyond laboratory parameters. Advanced imaging techniques, like cardiovascular magnetic resonance (CMR), with its ability to assess both myocardial infarction (through T2 mapping) and hepatic involvement, provide valuable insights into the systemic inflammatory response post-STEMI. Bergamaschi L. et al. documented an augmentation in hepatic T1 and extracellular volume, likely reflecting not only hepatic congestion secondary to right ventricular dysfunction, but also systemic and hepatic inflammation, which may further exacerbate heart failure progression. Therefore, these findings are valuable for better assessing high-risk post-STEMI patients, advising toward the integration of CMR in follow-up. In our future research, it would be interesting to analyze if combined myocardial T2 and hepatic T1 values provide additive prognostic value beyond traditional risk markers [84].

### Study Limitations

Including a relatively small number of analyzed patients, the lack of serial dosages to assess biomarker dynamics, and the impossibility of performing the routine 30-day checkup to evaluate the clinical and biological evolution represent the most import limitations of our study. Since the admitted patients originated from a wide geographical region, burdened by logistics difficulties (elderly and difficult to be transported at fixed schedules), we considered it more appropriate to perform only initial, acute-phase determinations. A multicenter approach would certainly improve the findings; by limiting certain exclusion criteria of this study, such a late presentation after symptom onset or previously administered fibrinolytic therapy for those patients out of the therapeutic window for a timely (<120 min) coronary angiography.

## 5. Conclusions

As compared with CCS patients, AMI patients exhibited greater mean values of inflammatory biomarkers, such as CRP, IL-6, IL-1β, and GDF-15. IL-1β was inversely correlated with the LVEF, while CRP, NT-proBNP, and uric acid levels were predictors of a severely reduced LVEF in the multivariate logistic regression analysis. Based on this pilot study, we outlined that NT-proBNP and IL-10 could be significant mortality predictors, while GDF-15 may be highly correlated with in-hospital death, in addition to cTn, NT-proBNP, and IL-10, which are reliable markers of increased mortality among patients with AMI. However, in the included patients, an elevated serum IL-6 concentration did not exert a negative prognostic role, nor was it correlated with overall mortality. The potential advantages of IL-1β over CRP as a prognostic biomarker and therapeutic target are represented by its location higher in the inflammatory cascade, as well as the existence of monoclonal antibodies directed against this cytokine. This promising perspective needs additional validation, together with the other two identified prognostic biomarkers IL-10 and GDF-15, as it could contribute to the risk stratification of patients with AMI.

Basically, in this study, we focused on highlighting the complex relationship between inflammation and coronary syndromes via the assessment of various biomarkers. Based on these findings, we also conceived several prediction models, including clinical and biological variables, that would be applicable in real-life practice. Nevertheless, further validation through extensive, multicentric studies are required for a comprehensive and reproducible conclusion.

## Figures and Tables

**Table 1:** Epidemiological and clinical data on admission.

	AMI (n = 105)	CCS (n = 40)	*t*-Test/*p* Value
**Age**	65.1 (12.5)	67.2 (10)	0.13
**Gender (male)**	73 (69.7%)	23 (57.5%)	0.17
**Temperature (°C)**	36.7 (0.34)	36.6 (0.27)	0.24
**Systolic blood pressure (mmHg)**	132.9 (24.2)	137.3 (17.8)	**0.02**
**Diastolic blood pressure (mmHg)**	80.5 (12.6)	83.0 (8.0)	**0.02**
**Heart rate (beats/minute)**	82.8 (18.8)	77.4 (13.3)	**0.03**
**Peripheral oxygen saturation (%)**	96.7 (3.5)	97.4 (4.6)	0.90
**Length of stay (days)**	6.10 (2.6)	2.73 (2.5)	**<0.001**
**Chest pain**	105 (100%)	34 (85%)	0.41
**Dyspnea**	40 (38.1%)	12 (30%)	0.46
**Orthopnea**	18 (17.1%)	2 (5%)	0.49
**Alcohol consumption**	29 (27.6%)	7 (17.5%)	0.21
**Smoking**	56 (53.3%)	16 (40%)	0.15
**Uric acid**	6.3 (1.9)	5.8 (1.7)	0.13
**Previous myocardial infarction**	6 (5.7%)	7 (17.5%)	0.08

Data are expressed as means (SD) or numbers and percentages (%); *p*-values were calculated using the *t*-test. AMI—Acute myocardial infarction, CCS—Chronic coronary syndrome. Significant values for *t*-test (*p* < 0.05) are marked with bold.

**Table 2:** Comparative analysis of cardiac and inflammatory biomarkers in AMI versus CCS.

Group Statistics					
	Group	N	Mean	Std. Deviation	*p*-Value
IL-1B	AMI	105	18.0930	57.51781	0.025
	CCS	40	4.4454	13.57680	
IL-6	AMI	105	15.4882	27.40994	0.028
	CCS	40	5.5334	11.01829	
IL-10	AMI	105	9.2936	20.42203	0.948
	CCS	40	9.5062	16.24560	
GDF-15	AMI	105	75.0531	85.94180	0.001
	CCS	40	25.0985	40.96897	
CRP	AMI	105	2.1814	3.18870	0.02
	CCS	40	0.5900	0.87894	
NT-proBNP	AMI	105	4029.75	5698.948	0.019
	CCS	40	1671.78	4289.596	
Troponin	AMI	105	6666.6476	10,231.67812	<0.001
	CCS	40	13.1058	8.10278	

**Table 3:** Biological predictors of severely reduced LVEF (LVEF < 30%).

Biological Parameters	*t*	df	Sig. (2-Tailed)	Mean Difference	Std. Error Difference	95% Confidence Interval of the Difference	
						Lower	Upper
**IL-1β**	−2.44	85.650	**0.017**	−18.19968	7.45604	−33.02266	−3.37670
**IL-6**	−1.37	101.07	0.172	−5.61804	4.08173	−13.71501	2.47894
**IL-10**	0.582	29.783	0.565	3.49594	6.00655	−8.77483	15.76670
**GDF-15**	1.045	34.008	0.303	23.16154	22.16333	−21.87938	68.20247
**CRP**	1.454	31.213	0.156	1.28227	0.88168	−0.51543	3.07998
**Ferritin**	0.495	33.641	0.624	19.522	39.477	−60.736	99.781
**Uric acid**	2.345	29.866	**0.026**	1.2653	0.5395	0.1632	2.3673
**NT-proBNP**	2.271	27.455	**0.031**	4026.918	1773.174	391.486	7662.349
**Troponin**	0.001	36.199	0.999	2.14250	2528.985	−5125.898	5130.18310

IL-1β—Interleukin 1β, IL-6—Interleukin 6, IL-10—Interleukin 10, GDF-15—Growth differentiation factor 15, CRP—C-reactive protein, NT-proBNP—N-terminal pro-brain natriuretic peptide. Significant values (*p* < 0.05) are marked with bold.

**Table 4:** LVEF < 30% correlations and biological parameters.

Biological Parameters	LVEF < 30%	
	*p*	r
**Total cholesterol**	0.502	−0.066
**LDL cholesterol**	0.506	−0.066
**HDL cholesterol**	0.858	0.018
**Triglycerides**	0.597	0.052
**Uric acid**	**0.003**	0.283
**Creatinine**	0.688	−0.040
**Urea**	**0.001**	0.324
**TSH**	0.577	−0.055
**FT4**	0.122	0.152
**Troponin**	0.999	0.000
**NT-proBNP**	**0.002**	0.302

LVEF—Left-ventricle ejection fraction, LDL—Low-density lipoprotein, HDL—High-density lipoprotein, TSH—Thyroid-stimulating hormone, FT4—Free thyroxine, NT-proBNP—N-terminal pro-brain natriuretic peptide. Significant values (*p* < 0.05) are marked with bold.

**Table 5:** Multivariate logistic regression model of predictive biomarkers for LVEF < 30%.

Variables in the Equation								
	B	S.E.	Wald	df	Sig.	Exp(B)	95% CI for Exp(B)	
							Lower	Upper
**IL-1β**	−0.024	0.021	1.291	1	0.256	0.976	0.937	1.017
**IL-6**	−0.074	0.033	5.024	1	**0.025**	0.929	0.871	0.991
**IL-10**	0.001	0.011	0.002	1	0.961	1.001	0.979	1.023
**GDF-15**	0.001	0.004	0.016	1	0.899	1.001	0.993	1.008
**CRP**	0.198	0.089	5.008	1	**0.025**	1.219	1.025	1.451
**Uric acid**	0.297	0.131	5.167	1	**0.023**	1.346	1.042	1.740
**NT-proBNP**	0.000	0.000	10.027	1	**0.002**	1.000	1.000	1.000
**Troponin**	0.000	0.000	0.004	1	0.948	1.000	1.000	1.000
**Constant**	−3.723	0.908	16.819	1	0.000	0.024		

IL-1β—Interleukin 1β, IL-6—Interleukin 6, IL-10—Interleukin 10, GDF-15—Growth differentiation factor 15, CRP—C-reactive protein, NT-proBNP—N-terminal pro-brain natriuretic peptide. Significant values (*p* < 0.05) are marked with bold.

**Table 6:** Hosmer–Lemeshow test for regression model validation.

Hosmer and Lemeshow Test			
Step	Chi-Square	df	Sig.
1	4.371	8	0.822

**Table 7:** Correlations between treatment/evolution parameters and inflammatory biomarkers.

Biomarker	Inotropic Support		Oxygen Therapy		Orotracheal Intubation		Intensive Care Unit		Death	
	*p*	r	*p*	r	*p*	r	*p*	r	*p*	r
**IL-1β**	0.355	−0.091	0.234	−0.117	0.303	−0.101	0.303	−0.101	0.646	−0.045
**IL-6**	0.162	0.137	0.128	0.149	0.283	0.106	0.283	0.106	0.096	0.163
**IL-10**	0.246	0.114	0.087	0.168	0.106	0.159	0.106	0.159	**0.044**	0.197
**CRP**	0.170	0.135	0.078	0.173	0.222	0.120	0.222	0.120	0.179	0.132

IL-1β—Interleukin 1β, IL-6—Interleukin 6, IL-10—Interleukin 10, CRP—C-reactive protein. The significant value (*p* < 0.05) is marked with bold.

**Table 8:** Correlations between biomarkers and in-hospital mortality by *t*-test.

	*t*	df	Sig. (2-Tailed)	Mean Difference	Std. Error Difference	95% Confidence Interval of the Difference	
						Lower	Upper
**IL-1β**	−2.331	78.306	**0.022**	−14.85447	6.37339	−27.54213	−2.16680
**IL-6**	1.372	4.616	0.233	14.36553	10.47207	−13.24213	41.97319
**IL-10**	2.971	103	**0.004**	26.81378	9.02510	8.91462	44.71294
**GDF-15**	1.691	4.407	0.159	66.21227	39.15941	−38.66732	171.09186
**CRP**	1.169	4.225	0.304	2.21820	1.89770	−2.94170	7.37810
**NT-proBNP**	2.657	4.087	**0.055**	12,251.450	4610.487	−443.032	24,945.932
**Troponin**	0.752	5.615	0.482	1980.46000	2633.49157	−4571.9558	8532.87586

IL-1β—Interleukin 1β, IL-6—Interleukin 6, IL-10—Interleukin 10, GDF-15—Growth differentiation factor 15, CRP—C-reactive protein, NT-proBNP—N-terminal pro-brain natriuretic peptide. Significant values (*p* < 0.05) are marked with bold.

**Table 9:** Correlations between biomarkers and in-hospital mortality.

Biomarkers	In-Hospital Death	
	*p*	r
**IL-1β**	0.654	−0.044
**IL-6**	0.095	0.164
**IL-10**	**0.042**	0.199
**GDF-15**	**0.040**	0.201
**NT-proBNP**	**0.005**	0.274
**Troponin**	0.092	0.165

IL-1β—Interleukin 1β, IL-6—Interleukin 6, IL-10—Interleukin 10, GDF-15—Growth differentiation factor 15, CRP—C-reactive protein, NT-proBNP—N-terminal pro-brain natriuretic peptide. Significant values (*p* < 0.05) are marked with bold.

**Table 10:** Multivariate logistic regression for mortality predictors.

	B	S.E.	Wald	*p*	Exp(B)
**NT-proBNP**	0.000	0.000	7.548	**0.006**	1.000
**GDF-15**	−0.004	0.006	0.343	0.558	0.996
**IL-10**	0.034	0.016	4.463	**0.035**	1.035

NT-proBNP—N-terminal pro-brain natriuretic peptide, GDF-15—Growth differentiation factor 15, IL-10—Interleukin 10. Significant values (*p* < 0.05) are marked with bold.

**Table 11:** Multimarker model in predicting mortality.

Model Summary										
Model	R	R Square	Adjusted R Square	Std. Error of the Estimate	Change Statistics					Durbin–Watson
					R Square Change	F Change	df1	df2	Sig. F Change	
1	0.409 ^a^	0.167	0.156	0.272	0.167	14.271	2	142	0.000	2.113

^a^ Predictors: (Constant), NT-proBNP, GDF-15; Dependent variable: Mortality; NT-proBNP—N-terminal pro-brain natriuretic peptide, GDF-15—Growth differentiation factor 15.

**Table 12:** Additional mortality predictors.

Biomarkers	Death	
	*p*	r
**LVEF < 30%**	**0.002**	0.295
**NYHA functional class**	**0.014**	0.240
**Hospitalization length**	**0.029**	−0.213
**Smoking**	0.545	−0.060
**Oxygen therapy**	0.810	−0.024
**Inotropic support**	**<0.001**	0.441
**Orotracheal intubation**	**<0.001**	0.837

LVEF—Left-ventricle ejection fraction. NYHA—New York Heart Association. Significant values (*p* < 0.05) are marked with bold.

**Table 13:** Multivariate linear regression—prediction score for non-biomarker death.

Model	R	R Square	Adjusted R Square	Std. Error of the Estimate	Change Statistics					Durbin–Watson
					R Square Change	F Change	df1	df2	Sig. F Change	
1	0.837 ^a^	0.700	0.697	0.118	0.700	240.333	1	103	**<0.001**	
2	0.846 ^b^	0.715	0.709	0.115	0.015	5.391	1	102	**0.022**	2.246

^a^ Predictors: (Constant), orotracheal intubation, ^b^ Predictors: (Constant), orotracheal intubation, LVEF < 30%, Dependent Variable: Death. LVEF—left ventricle ejection fraction. Significant values (*p* < 0.05) are marked with bold.

**Table 14:** Multivariate linear regression for mortality predictors.

Model	R	R Square	Adjusted R Square	SE	R Square Change	F Change	df1	df2	Sig. F change	Durbin– Watson
1	0.837 ^a^	0.700	0.697	0.118	0.700	240.33	1	103	**<0.001**	
2	0.853 ^b^	0.728	0.723	0.113	0.028	10.540	1	102	**0.002**	2.339

^a^ Predictors: (Constant), orotracheal intubation, ^b^ Predictors: (Constant), orotracheal intubation, NT-proBNP, Dependent variable: Mortality. Significant values (*p* < 0.05) are marked with bold.

**Table 15:** AUC and the detailed significance of biomarkers in predicting mortality.

Area Under the ROC Curve					
Test Result Variable(s)	Area	Std. Error ^a^	Asymptotic Sig. ^b^	Asymptotic 95% Confidence Interval	
				Lower Bound	Upper Bound
**IL-1β**	0.440	0.149	0.688	0.148	0.732
**IL-6**	0.722	0.137	0.104	0.454	0.990
**IL-10**	0.770	0.080	**0.001**	0.613	0.927
**GDF-15**	0.772	0.081	**0.001**	0.613	0.931
**NT-proBNP**	0.872	0.088	**<0.001**	0.700	1.044
**Troponin**	0.724	0.085	**0.008**	0.557	0.891

^a^ Under the nonparametric assumption, ^b^ Null hypothesis: true area = 0.5. Significant values (*p* < 0.05) are marked with bold.

**Table 16:** Highlight section concerning analyzed biomarkers.

IL-1β stands out for its significant prognostic role as it may further contribute to a more comprehensive approach to heart failure with reduced left-ventricle ejection fraction of ischemic etiology.
IL-6 did not exhibited a diagnostic or prognostic role in patients with acute myocardial infarction.
IL-10 reflects a pronounced inflammatory state in patients with acute myocardial infarction, plays a crucial role in counteracting the inflammatory response, and has superior predictive value in terms of fatal prognosis compared with other modern biomarkers.
GDF-15 is a surrogate biomarker involved in inflammation, heart failure, and renal dysfunction in patients with acute myocardial infarction.

## Data Availability

Data are entirely contained within the article.

## Supplementary Materials

The following supporting information can be downloaded at: https://www.mdpi.com/article/10.3390/diagnostics15101211/s1, Table S1: The correlations between biomarkers and culprit (AMI) or stented (CCS) lesions.
