# Supplementary material for: Integrating the Interleukins in the Biomarker Panel for the Diagnosis and Prognosis of Patients with Acute Coronary Syndromes: Unraveling a Multifaceted Conundrum
Source: Diagnostics (Basel). 2025 May 11;15(10):1211. doi: 10.3390/diagnostics15101211 (PMC12110678; doi:10.3390/diagnostics15101211)
Supplement: Supplementary file 1 [file diagnostics-15-01211-s001.zip › diagnostics-3600711-supplementary.pdf]

Supplementary Materials

Table S1. The correlations between biomarkers and culprit (AMI) or stented (CCS) lesions.

Dependent variable	Culprit lesion (AMI) Stented lesion (CCS) Location	Mean Difference (I-J)	Std. Error	Sig.	95% Confidence Interval	
					Lower Bound	Upper Bound
IL-1β	ADA/LM	-11.08157	16.39566	.874	-52.7786	30.6155
		1.30587	12.89668	.999	-30.3140	32.9258
	LCX	11.08157	16.39566	.874	-30.6155	52.7786
		12.38744	18.43406	.876	-33.6228	58.3977
	RCA	-1.30587	12.89668	.999	-32.9258	30.3140
		-12.38744	18.43406	.876	-58.3977	33.6228
IL-6	ADA/LM	-2.81978	6.99172	.969	-20.3296	14.6900
		-1.73831	6.22578	.989	-16.9459	13.4693
	LCX	2.81978	6.99172	.969	-14.6900	20.3296
		1.08147	7.63453	.999	-17.8791	20.0420
	RCA	1.73831	6.22578	.989	-13.4693	16.9459
		-1.08147	7.63453	.999	-20.0420	17.8791
IL-10	ADA/LM	1.48786	5.11743	.988	-11.0838	14.0595
		5.90842	4.12633	.397	-4.1930	16.0099
	LCX	-1.48786	5.11743	.988	-14.0595	11.0838
		4.42056	3.79437	.576	-5.2274	14.0685
	RCA	-5.90842	4.12633	.397	-16.0099	4.1930
		-4.42056	3.79437	.576	-14.0685	5.2274
GDF-15	ADA/LM	-.30706	19.00623	1.000	-48.1085	47.4943
		-30.24040	20.75074	.384	-81.2957	20.8149
	LCX	.30706	19.00623	1.000	-47.4943	48.1085
		-29.93334	24.53328	.535	-90.4353	30.5686
	RCA	30.24040	20.75074	.384	-20.8149	81.2957
		29.93334	24.53328	.535	-30.5686	90.4353
CRP	ADA/LM	-1.49084	.93510	.322	-3.9000	.9184
		-.59983	.72295	.791	-2.3861	1.1865
	LCX	1.49084	.93510	.322	-.9184	3.9000
		.89101	1.11164	.808	-1.8816	3.6636
	RCA	.59983	.72295	.791	-1.1865	2.3861
		-.89101	1.11164	.808	-3.6636	1.8816
